# From Histopathology to High-Resolution Ultrasound Imaging of Skin Scars

**DOI:** 10.3390/diagnostics13243629

**Published:** 2023-12-08

**Authors:** Vincenzo Ricci, Giulio Cocco, Danilo Donati, Giacomo Farì, Ke-Vin Chang, Levent Özçakar

**Affiliations:** 1 Physical and Rehabilitation Medicine Unit, Luigi Sacco University Hospital, ASST Fatebenefratelli-Sacco, 20157 Milan, Italy; 2 Department of Neuroscience, Imaging and Clinical Sciences, “G. D’Annunzio” University, 66100 Chieti, Italy; cocco.giulio@gmail.com; 3 Clinical and Experimental Medicine PhD Program, University of Modena and Reggio Emilia, 41121 Modena, Italy; da.donati@yahoo.it; 4 Department of Biological and Environmental Sciences and Technologies (DiSTeBA), Università del Salento, 73100 Lecce, Italy; dr.giacomofari@gmail.com; 5 Department of Physical Medicine and Rehabilitation, National Taiwan University Hospital, Bei-Hu Branch, Taipei 10617, Taiwan; kvchang011@gmail.com; 6 Department of Physical and Rehabilitation Medicine, Hacettepe University Medical School, 06100 Ankara, Turkey; lozcakar@yahoo.com

**Keywords:** epidermis, dermis, injury, histology, ultrasonography

## Abstract

Nowadays, modern ultrasound machines and high-frequency transducers allow us to accurately assess the superficial soft tissues of the human body. In this sense, sonographic evaluation of the skin and related pathologies is progressively growing in the pertinent literature. To the best of our knowledge, a standardized sonographic protocol focused on the assessment of pathological skin scars is still lacking. As such, the main purpose of the present study was to propose a technical guide to sonographically assess skin scars in the daily practice of clinicians—starting from knowledge on their histopathological features. In order to standardize the ultrasound examination, a superficial-to-deep, layer-by-layer approach has been proposed to optimize its reproducibility and to promote a common language among the different healthcare providers.

## 1. Introduction

The superficial soft tissues of the human body such as skin and subcutis can be accurately visualized by using modern ultrasound (US) equipment and high-frequency transducers [1]. Indeed, the superior lateral spatial resolution of high-end machines allows us to differentiate several histological layers—i.e., epidermis, dermis, dermo-hypodermal interface (DHI), and the subcutaneous tissue [2,3]. Moreover, adjusting the technical settings for depth, focus, and frequency range can further optimize the sonographic visualization of the aforementioned tissues [4]. In this aspect, the ever-increasing capacity of US imaging to assess different histological compartments of the (superficial) soft tissues has led to the modern definition of sono-histology. Technically, a large amount of gel is necessary to avoid unintentional compression of the skin with the US transducer preserving the native shape, thickness, and vascularization of the superficial soft tissues. The aforementioned suspension technique—coupled with optimal settings for power Doppler imaging (e.g., pulse repetition frequency)—is paramount to accurately visualize the small-size and low-flow vascular elements [5,6]. Indeed, two main lymphovascular plexuses are located within the dermis, a superficial and a deep one. The superficial plexus is composed of terminal arterioles, capillaries, and postcapillary venules arranged in a candelabra-like loop system inside the dermal papillae, with an ascending arterial branch and a descending venous branch [5]. The deep plexus is located in between the reticular dermis and the subcutaneous tissue and presents small arteries and terminal arterioles anastomosed with the overlying superficial lymphovascular plexus via vertically oriented blood and lymphatic vessels [5]. Moreover, a complex network of superficial veins is located between the DHI and the deep fascia—also known as the epifascial venous system [5]. Its superficial portion runs within the DHI receiving the venous drainage from the dermis and subcutis; likewise, its deep portion is mainly located at the level of the deep fascia [5].

Despite the above quoted technological advances of US machines and their wide application in dermatology, the literature on the sonographic assessment of skin scars (SSs) is quite poor. Bessonart et al., in 2005, evaluated six pathological SSs from five patients using a 20 MHz US probe and compared them with the healthy contralateral sides [7]. The authors described the echotexture of the SSs as hypoechoic compared with the surrounding highly echogenic dermal tissue—i.e., “echo-poor structures”. Considering the presence of high amount of connective tissue and collagen inside the SSs (which should produce a hyperechoic image), they explained the paradoxical effect of a hypoechoic sonographic pattern as related to the presence of abundant water-binding molecules such as proteoglycans and glycoproteins [7].

Normally, wound healing involves a series of biochemical processes that progressively lead to the formation of a normotrophic scar that is relatively flat, thin, and inconspicuous [8]. In patients with excessive wound healing, abnormal scars may develop with stratum corneum barrier dysfunction and upregulation of epidermal differentiation and proliferation markers, e.g., hypertrophic scar (HS) and keloid (K) [8,9]. Both of them involve excessive collagen deposition; but K, unlike HS, exhibits fibrotic tissue that extends beyond the boundaries of the original wound (i.e., invasive horizontal growth) and does not regress during time [10]. Lobos et al., in 2017, defined K as hypoechoic and/or heterogeneous thickening of the dermis that displaces the epidermis upward, with(out) a laminar pattern and with(out) extension to the deeper layers [11].

The main purpose of the present research was, starting from the histological features of SSs, to propose a simple, ready-to-use, layer-by-layer sonographic protocol for the daily clinical practice. We strongly believe that the present standardized approach will represent a step further to optimize their prompt management.

## 2. Materials and Methods

The authors planned a 3-step workflow as described below:

1st Phase: An extensive review of the scientific literature on histopathological features of SSs was performed. The research mainly focused on their macroscopical aspects such as shape, contracture, local infiltration, and vascularization (rather than their immunohistochemical features)—as the former may present corresponding sonographic findings. PubMed and Web of Science were searched using the following keywords: “skin”, “scar”, “histology”, “histopathology”, “hypertrophic scar”, and “keloid”.

2nd Phase: Using high-frequency US transducers (i18LX5 with a range of frequency 4.8–18 MHz and an i33LX9 matrix linear transducer with a center frequency of 30 MHz) and high-end machines (Canon Aplio i800, Canon Medical Systems, Shimoishigami, Otawara-Shi, Japan), different types of pathological SSs were sonographically evaluated. A physician with more than 15 years of experience in US imaging of soft tissues collected the images in 8 months. The average exam execution time was 30 min, and informed consent was acquired from each patient involved in the present research. The patients were referred for the US examination by general practitioners and dermatologists, and a total of 15 SSs were evaluated. The US examination was performed using a distal gripping technique, gently placing the ulnar side of the examiner’s hand over the skin of the patient to reduce the compression of superficial soft tissues as much as possible—preserving their native shape. The depth was adjusted to optimize the visualization of superficial soft tissues in a field of view of 1–2 cm beneath the skin surface. A single focal zone was used and positioned at the level of the dermis. The overall gain was set to optimize the contrast resolution between the hyperechogenicity of the dermis and the hypoechogenicity of the subcutaneous tissue. A layer-by-layer US assessment was performed as reported in Table 1. The epidermis, dermis, DHI, and subcutaneous tissue are the histological layers accurately checked in order to guarantee a standardized and reproducible description of the SSs. Of note, the SSs that were sonographically evaluated have not undergone a selective biopsy; therefore, a direct cross-match between the sonographic and histological findings has not been performed in the present study. All the data reported in the present research have been used retrospectively, analyzing the images of the database of the Department.

3rd Phase: Starting with the histopathological features and collecting their sonographic patterns, a standardized US protocol was proposed in this manuscript. Considering the methodology applied, i.e., without a head-to-head comparison of histopathological and sonographic findings, the aim of this research was not to provide a protocol to perform the sonographic differential diagnosis between HS and K. Instead, the authors intended to establish a practical guide describing “how” to perform the sonographic evaluation of pathological SSs in daily practice.

## 3. Results and Discussion

Taking into account the peculiar nature of the present manuscript, the authors combined the results and discussion to provide a coupled description of the histopathological and high-resolution US findings of pathological SSs.

### 3.1. Histopathology of SSs

The skin presents three different histological layers—the epidermis, dermis (papillary and reticular), and subcutaneous tissue. Although the histologic appearance of the skin may significantly vary, depending on the anatomical site, and the age of the patient may largely influence the histological architecture of the skin, some basic histo-anatomical landmarks can be recognized. The epidermis is a stratified and keratinizing squamous epithelium made of keratinocytes, melanocytes, Merkel, Langerhans, and Toker cells [12,13]. The papillary dermis shows as a loose meshwork composed of thin and poorly organized type III collagen coupled with an abundant ground substance rich in water, glycosaminoglycans, and acid mucopolysaccharides [12,13]. The reticular dermis presents multiple layers of well-organized thick bundles of (mainly type I) collagens and thick elastic fibers aligned parallel to the skin surface and randomly oriented in different spatial directions [14,15]. Lastly, the subcutaneous tissue (subcutis) is characterized by mature adipose tissue arranged into multiple fat lobules separated from each other by thick bands of connective tissue also known as interlobular septa or fibrous scaffold [12,13].

The histologic exam of SSs typically shows a non-specific dermal fibroblastic and myo-fibroblastic proliferation with nodular and/or multinodular growth pattern, epidermal atrophy, and deposition of hyalinized collagen bundles [12,16,17,18]. An inflammatory infiltrate is usually detected within the scarring tissue, whose quantitative and qualitative features largely depend on the pathologic condition and the phase of the healing process. Indeed, in the early phase, the SS usually presents an abundant and mixed (lymphocytes, macrophages, granulocytes, plasmacells, etc.) inflammatory infiltrate. After the first 24–36 h of the inflammatory phase, the formation of lymphatic and vascular neo-channels coupled with mitosis of fibroblasts and endothelial and mesenchymal cells define the proliferative phase with an intense deposition of collagen fibers [12,16]. In the subsequent remodeling phase, the vascular network inside the scarring tissue becomes progressively reduced. Collagen fibers adjust their spatial orientation in accordance with the lines of forces, and the skin surface shows a reepithelization. In this sense, in the later phases, the SSs are mainly fibrotic with scattered interstitial lymphocytes [12,16,17,18]. The histologic composition of the inflammatory infiltrate may also vary depending on the pathologic condition, ranging from a non-specific and interstitial lympho-plasmacellular infiltrate to a non-necrotizing granulomatous reaction with multinucleated giant cells—i.e., stitches, foreign body reaction, and destruction of follicular structures with extravasation of keratin/hair shafts into the dermis [12,16,17,18].

In some patients, SSs display an exuberant and exaggerated growth pattern, raising the clinical suspicion of HS and/or K. Both are considered as clonal benign proliferations/tumors mediated by hyperactivation of the wound-healing pathways (TGF-β, Wnt/β-catenin, etc.) [12,16,19]. Some authors consider the amount of current evidence as insufficient to distinguish HS and K as separate entities. In this sense, they can represent the same pathological process with different quantitative features—less pronounced in HS and more pronounced in K [20,21]. Morphologically, K is prone to develop a horizontal growth extending beyond the original wound boundaries, unlike HS, which is commonly confined inside the original wound edges (Table 2) [12,16,22]. Clinically, K is less frequent compared with HS, and it is more prone to local recurrence (causing aesthetic problems) and does not regress over time [23,24]. As regard the size, HS commonly measures less than 1 cm in thickness/width; meanwhile, K can develop in variable sizes, even reaching large dimensions [23,24].

**Figure 1:**
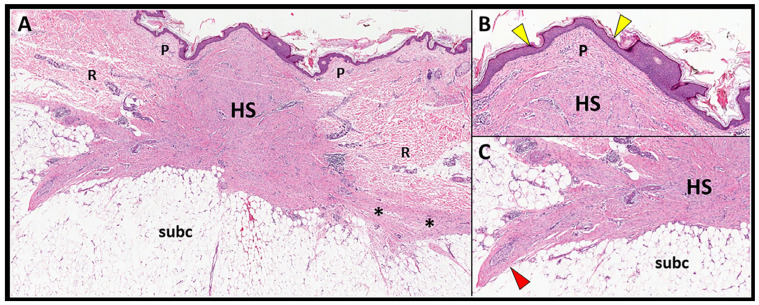
Histopathological features of HS. The histological sample (**A**) shows a hypertrophic scar (HS) involving the dermis throughout its entire thickness and reaching the dermo-hypodermal interface (black asterisks). Magnifications (**B**,**C**) clearly show the flattened epidermis (yellow arrowheads), the scarring involvement of the papillary dermis (P), and the spiculated boundaries (red arrowhead) penetrating and deforming the underlying subcutaneous tissue (subc). R: reticular dermis.

The histologic exam may play a key role in distinguishing HS and K. Namely, flattened epidermis, scarring involvement of the papillary dermis, vertically oriented blood vessels, and increased α-smooth muscle actin expression are histologic features more commonly detected in HS than K (Figure 1) [16,17,18].

Notably, HS (compared with K) may develop excessive contracture, mainly promoted by the (myo)fibroblastic cells expressing smooth-muscle actin—retaining contractile proprieties, abnormally tensioning and stretching the surrounding histological elements [9]. The mechanical distortion of the soft tissues surrounding HS may have a key role in the development of neuropathic pain by directly stimulating the local nociceptive fibers. Indeed, more than 1 million myelinated and nonmyelinated free nerve endings (responsible for the perception of temperature, itch, and pain) widely branch within the epidermis and dermis, presenting a tuft-like arrangement [16].

**Figure 2:**
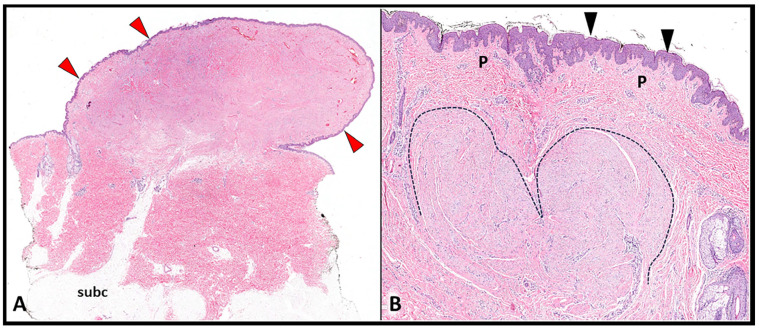
Histopathological features of K. The histological sample (**A**) shows how the keloid progressively develops a horizontal growth (red arrowheads) extending beyond the edges of the original wound. Magnification (**B**) confirms the normal thickness of the epidermis (black arrowheads), sparing of the papillary dermis (P), and the tongue-like advancing edge of the keloid (black dotted line) from the deep layers of the reticular dermis. subc: subcutaneous tissue.

By contrast, K presents a peculiar histological component known as keloidal collagen. The latter is characterized by thickened, hyalinized, and glassy collagen bundles, with a chaotic spatial arrangement, and surrounded by a mucinous ground substance. Moreover, unlike HS, the peripheral edge of scarring tissue presents a shape similar to a tongue in K (Figure 2)—coupled with a horizontal fibrous band located in the upper compartment of the reticular dermis [12,16,17,18].

Lastly, the presence and distribution of the vascular network seem to be a key histological feature for the differential diagnosis between K and HS. Notably, arborizing, curvilinear, and comma-shaped vascular structures are present in the majority of Ks and more rarely in HSs [25].

### 3.2. Sonography of SSs

First and foremost, in order to avoid misinterpretation of the US findings, the most suitable timing to perform the assessment of the skin scar should be considered.

**Figure 3:**
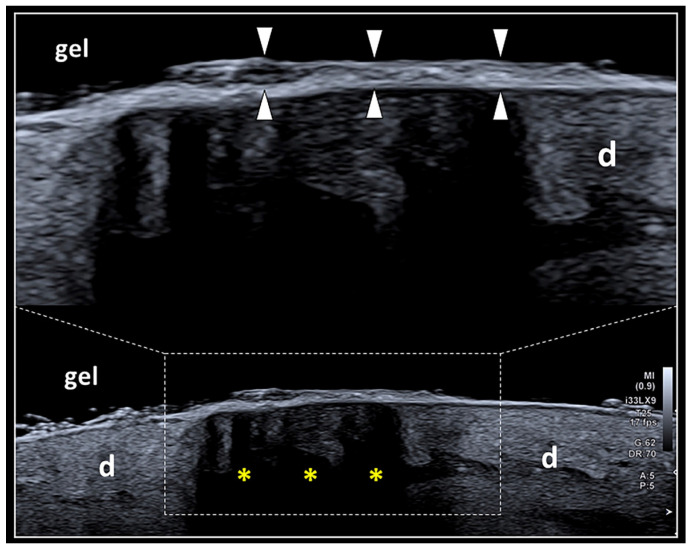
The “deep masking effect”. Massive deposition of keratin laminae over the surface of the epidermis (white arrowheads) may generate a hyper-reflective interface reducing the penetration of the US beam within the deep layers and with the development of dark shadows (yellow asterisks) that mask the inner echotexture of the dermis (d). The “deep masking effect” can be physically considered very similar to the artifact of partial acoustic shadowing.

In the early phase of the healing process of skin wounds (7–12 days), the US examination should be avoided due to two main issues: the potential infectious risk related to the discontinuity of the epidermis and the so-called “deep masking effect”. The latter is a technical pitfall related to the massive thickening of the stratum corneum of the epidermis, which highly reflects the US beam preventing the visualization of the deep layers (Figure 3) [26]. It can be considered quite similar to the classical artifact of the posterior acoustic shadowing, commonly generated by hyper-reflective surfaces of the human body, e.g., the cortical bone or calcific deposits [27]. In this case, a practical trick is represented by the slight shift/tilt of the transducer to orient the US beam through the lateral aspect of the scar, circumventing the acoustic shadowing and improving the visibility of the deep tissues. Progressively, in the sub-acute (>15 days) or chronic (>30 days) phase of the healing process of skin wounds, the thickness of the stratum corneum and the spatial arrangement of the keratin laminae cover the scar change, allowing the US beam to penetrate toward the deeper tissue planes.

#### 3.2.1. Epidermis

The epidermis appears as a thin hyperechoic line on US imaging [28,29]. The high difference in the acoustic impedance between the gel and the epithelium, the presence of a superficial keratinized stratum corneum, and its compact architecture with tight junctions are the main physical and histological reasons for the high echogenicity of this layer [26]. Despite the recent advances in US machines, clear sonographic visualization of epidermal lesions, especially those that measure less than 0.1 mm in depth, cannot be accurately guaranteed [30]. The histological interface between the epidermis and dermis is known as the dermal–epidermal junction. Histologically, this layer appears wavy due to the presence of dermal papillae, but, sonographically, it presents with a linear arrangement due to the very small size of the papillae—not detectable with any of the high-frequency US transducers used for this research [26]. This transitional interface is made of a basement membrane and a complex mixture of numerous substances such as glycosaminoglycans, laminin, and different collagen isoforms [12,13].

**Figure 4:**
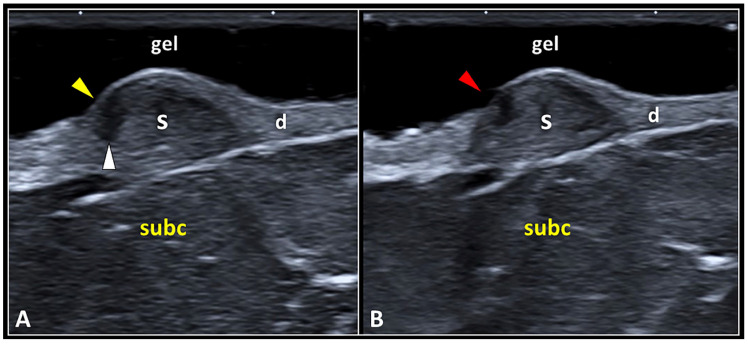
The dermo-epidermal dissociation. Retraction of the scar tissue (S) may generate a gap (white arrowhead) in between the epidermal (yellow arrowhead) and dermal (d) layers, i.e., dermo-epidermal dissociation (**A**). This condition can evolve in a focal discontinuity of the epidermis (red arrowhead), probably due to a local hypo-vascularization, with superficial exposure of the underlying scarring tissue (**B**). subc: subcutaneous tissue.

Normally, the hyperechoic epidermis covers the underlying hypoechoic scar tissue located in the dermis. In some patients, the progressive remodeling of the scar may lead to its detachment from the overlying epidermis—i.e., dermo-epidermal dissociation. This condition can progressively evolve into a focal disruption of the epidermis with exposure of the underlying scar tissue (Figure 4). Sonographically, a focal discontinuity of the hyperechoic line representing the epidermis can be observed coupled with a hypo/anechoic focus within the underlying dermal tissue.

Focal injury of the epidermal layer overlying the site of dermo-epidermal dissociation is most likely related to local hypoperfusion—with mechanical disruption of the hemidesmosomal adhesion proteins at their attachment to the underlying laminae (lucida and densa). Yet, as previously mentioned, there are terminal arterioles, capillaries, and postcapillary venules arranged in a “candelabra-like loop system” located into each dermal papilla, with ascending arterial and descending venous branches [26,31]. The authors strongly suggest accurately reporting the aforementioned sonographic patterns of the epidermis in patients with skin scars—to plan a suitable treatment to avoid infection of the superficial soft tissues. For instance, special bandages to protect the scarring tissue, and/or topical pharmacological agents to create a protective film over the focal discontinuity of the epidermis can be used. Likewise, in patients with a clinical suspicion of epidermal interruption (after the physical examination), a sterile gel should be adopted to prepare the soft standing pad over the skin scar before the US assessment.

#### 3.2.2. Dermis

**Figure 5:**
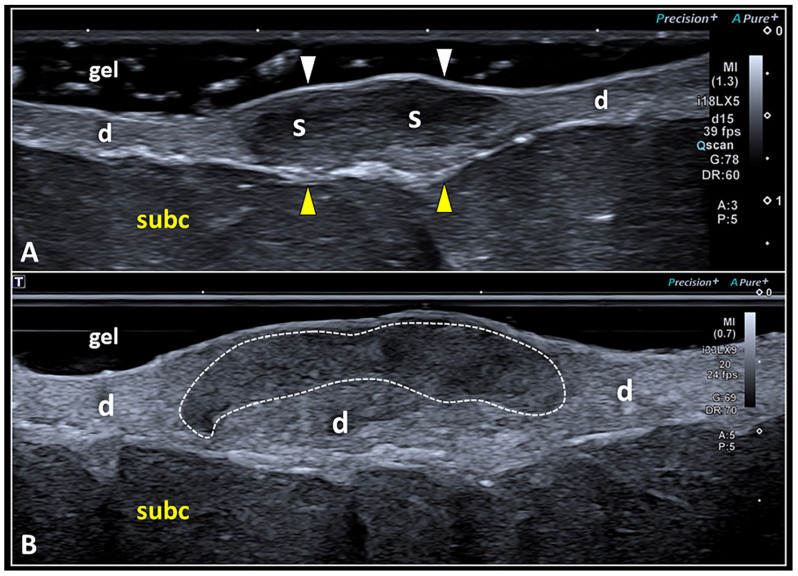
High-frequency US imaging of SSs. Hypoechoic scarring tissue (S) may involve the dermis (d) in its entire thickness, displacing the overlying epidermis (white arrowheads) and the underlying dermo-hypodermal interface (yellow arrowheads) (**A**). Using the very high-frequency transducer, a scar (white dotted line) selectively involving the most superficial portion of the dermis (d) and preserving its deep component can be visualized using the suspension technique (**B**). subc: subcutaneous tissue.

The dermis is a ribbon-like band less echogenic than the overlying epidermis and more echogenic than the underlying subcutaneous tissue [26,28]. Its echotexture is mainly related to the presence of (type I and III) collagen fibers and abundant ground substance that is highly hydrated [26,28]. Indeed, the latter is rich in water-binding molecules such as glycosaminoglycans and mucopolysaccharides [12,13]. The most common sonographic aspect of the skin scar is a focal hypoechogenicity of the dermis due to the presence of scarring tissue (Figure 5).

As previously mentioned, despite the high concentration of collagen fibers inside the scar that generate additional acoustic interfaces compared with the physiological dermis, it is paradoxically hypoechoic [7]. This unexpected sonographic texture seems to be related to the presence of water molecules bound to the glycosaminoglycans and proteoglycans, and the fluids widely distributed inside the extracellular matrix of skin scars [7,32,33]. Nowadays, high-frequency US transducers allow a very detailed evaluation of the spatial location of the skin scar within the dermis [34]. Indeed, the fibrotic tissue can involve the entire thickness of the dermal layer or can selectively involve its superficial or deep portion (Figure 5).

**Figure 6:**
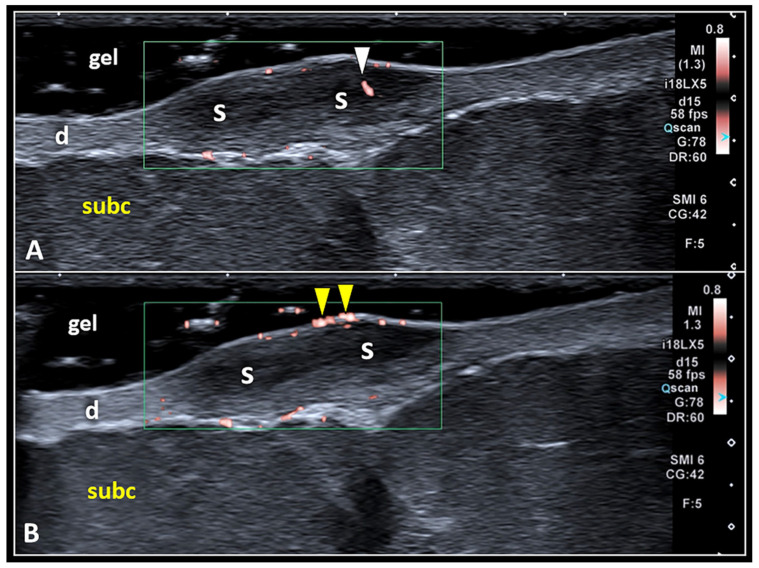
Pitfalls in Doppler imaging of SS. Using a large amount of gel (i.e., the suspension technique) and fully including the scarring tissue (S) inside the Doppler box, it is paramount to accurately differentiate the real vascular signals (white arrowhead) inside the scar (**A**) from the ‘fake’ ones (yellow arrowheads) generated by the acoustic interface between the epidermis and the gel (**B**). d: dermis, subc: subcutaneous tissue.

The edges of the scar can be ill-defined and poorly recognizable with a progressive passage from the scar to the normal dermis (coarse transition). They can also be well defined with a clear interface between the scar’s boundaries and the normal dermal tissue. The color/power Doppler assessment of scarring tissue requires accurate adjustment of technical parameters such as the Doppler gain/box and pulse repetition frequency [5,35]. The area of interest should be large enough to cover the entire scar; however, we suggest avoiding excessive sizes, which might decrease Doppler sensitivity in detecting low flows pertaining to small-size vascular elements (Figure 6) [36,37].

Using color Doppler US and comparing with clinical scoring, Lobos et al., in 2017, published a retrospective study describing the vascular patterns of 42 clinically diagnosed Ks [11]. They proposed a color Doppler US grading of Ks (inactive in the absence of vascular signals, low and high in the presence of vessels) and concluded that clinical evaluation alone can underestimate the activity in Ks [11]. Also, using high-end machines (Canon Aplio i800, Canon Medical Systems, Shimoishigami, Otawara-Shi, Japan) and high-frequency US transducers (i18LX5, i33LX9), we can identify rare and small-size vascular signals inside the scarring tissue. We speculate that this finding may be related to the poor presence of vascular elements within a skin scar in the inactive phase (absence of inflammatory and remodeling phenomena). Of note, in clinical practice, the sonographic appearance of well-defined hyperechoic scarring tissue without inner neo-vessels usually defines a “mature” skin scar that has completed the active remodeling process. In such cases, if clinically indicated, manual treatments such as stretching and massages and physical agents such as superficial heat can be applied to the fibrotic tissue to improve the skin elasticity and the gliding of superficial soft tissues.

Likewise, artifactual flow signals at the gel–epidermis interface should not be misinterpreted as real vascular signals of the scarring tissue [27]. Indeed, fake vascular signals generated at the acoustic interface between the two different histological compartments are quite frequent during the musculoskeletal US and can be considered as a pitfall, especially for beginners. In this sense, minor shifts of the transducer over the target area or tiny changes in the pressure applied over the gel pad are very often tricks sufficient to promote the disappearance of these artifacts.

#### 3.2.3. Dermo-Hypodermal Interface and Subcutaneous Tissue

The DHI can be sonographically visualized as a thin hyperechoic line between the dermis and the underlying subcutaneous tissue [35]. Likewise, the subcutaneous tissue (subcutis) presents multiple hypoechoic fat lobules mechanically stabilized by a network-shaped hyperechoic fibrous scaffold [35]. The latter is mainly composed of type IV and VII dense/thick collagen bundles, elastic fibers, and fibroblasts; and it connects the dermis to the deeper anatomic structures providing stability and elasticity to the subcutis [38,39]. Large blood and lymphatic vessels travel within the interlobular septae of the fibrous scaffold branching into smaller elements that penetrate both into the intercellular matrix of the adipose lobules of subcutis and into the dermis to anastomose with its deep lymphovascular plexus [12,13].

The scarring tissue originating from the dermal layer may interact with the underlying structures in an expansive or infiltrative pattern (Table 3).

**Figure 7:**
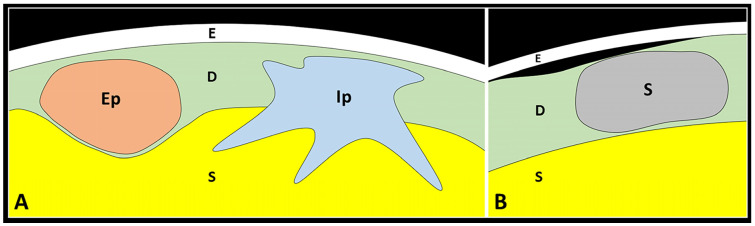
Dermo-hypodermal and dermo-epidermal interfaces. The skin scar may present an expansive pattern (Ep) compressing the underlying subcutaneous fat tissue (S) or an infiltrative pattern (Ip) with distal expansions that penetrate inside the subcutaneous layer (S) (**A**). Detachment of the epidermis (E) from the underlying dermis (D) can occur near the scarring tissue (S)—i.e., the dermo-epidermal dissociation (**B**).

The expansive pattern is commonly characterized by a globular shaped scar that compresses the fat lobules of the underlying subcutis deflecting but preserving the hyperechoic DHI (Figure 7).

**Figure 8:**
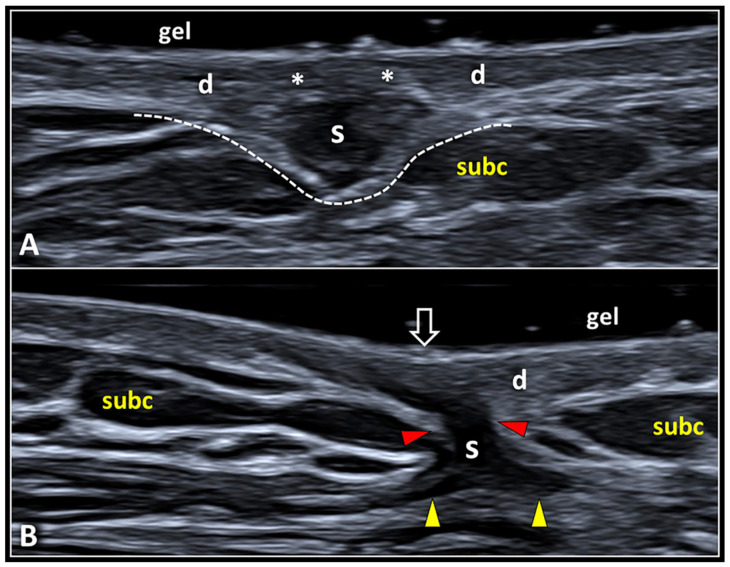
Expansive vs. infiltrative pattern of SSs. A globular shaped scar (S) originating from the deep portion of the dermis (d) compresses the underlying subcutaneous fat tissue (subc) preserving the histological continuity of the dermo-hypodermal interface (white dotted line) (**A**). Note that the superficial portion of the dermis (white asterisks) is not involved in the fibrotic involution (**A**). An irregular shaped scarring tissue (S) interrupts the dermo-hypodermal interface (red arrowheads) and shows distal expansions (yellow arrowheads) infiltrating the subcutis (**B**). Interestingly, this type of scar (S) shows a contractile behavior mechanically tensioning the overlying epidermis (void arrow) and distorting the surrounding subcutaneous tissue (subc) (**B**).

In this sense, the scar tissue with an expansive pattern shows a mass effect pushing the surrounding soft tissues without retraction. In some patients, the epidermis and the superficial portion of the dermis may develop a complete histological healing “hiding” the scar in the deeper layers (Figure 8). In these cases, inspection during the traditional physical examination may underestimate the real size/extension of the scar tissue whereby US examination becomes necessary to identify and measure the skin scar. Of note, the continuity of hyperechoic DHI must be confirmed for the entire extension of the skin scar, considering the fact that the infiltrative process toward the underlying subcutis may selectively involve the central or more lateral (peripheral) portion of the scarring tissue.

The infiltrative pattern, instead, usually presents a scarring tissue irregular in shape that interrupts the DHI—penetrating within the subcutaneous fat and mechanically distorting the surrounding tissue. The hypoechoic scar crosses the hyperechoic DHI and generates thin expansions insinuating in between the adipocytes of the subcutis (Figure 7). The sonographic pattern obtained with high-frequency US transducers accurately matches the aforementioned histopathological features of HS, with fibrotic digitations advancing within the subcutaneous tissue (Figure 1). In these cases, the concave deflection of the epidermis and the spatial disorganization of the fat lobules and fibrotic scaffold of subcutis can be considered as indirect sonographic signs of scar retraction. If clinically indicated, in patients with the infiltrative pattern, a second-line diagnostic tool such as biopsy may be necessary to accurately define the histopathological features of the mass.

## 4. Conclusions

Nowadays, by using high-end machines and high-frequency US transducers, a detailed sonographic assessment of the SSs can be performed in clinical practice. To this end, the present US protocol is intended to provide a simple and ready-to-use practical guide in the daily practice of physicians to perform a standardized (layer-by-layer) evaluation. Last but not least, using high-resolution B-mode and high-sensitive color/power Doppler imaging, the differential diagnosis between HS and K would be better interpreted.

## Figures and Tables

**Table 1:** Sonographic approach to the SSs.

Histological Compartment	Ultrasound Findings
Epidermis	Continuous, interrupted, hyper-reflective ^§^ +/− dermo-epidermal dissociation
Dermis	Thickness, echogenicity, and shape * of the scar tissue +/− vascular signals on CD/PD
DHI	Continuous, interrupted, retracted
Subcutaneous tissue	Infiltrative pattern, compressive pattern

^§^ Deep masking effect; * flat, globular, irregular-in-shape; CD: color Doppler; PD: power doppler; DHI: dermo-hypodermal interface.

**Table 2:** Hypertrophic Scar vs. Keloid.

Hypertrophic Scar	Keloid
High incidence	Low incidence
No association with skin color	Association with darker skin
Confined within original wound boundaries	Extension beyond the original wound boundaries *
Usually <1 cm thick/wide	Variable size
Associated with contracture	Not associated with contracture

* Horizontal growth.

**Table 3:** Expansive vs. Infiltrative Pattern.

Expansive Pattern	Infiltrative Pattern
Scar flat or globular in shape	Scar irregular in shape *
Compression of the SUBC	Infiltration of the SUBC
Continuous DHI	Interrupted DHI
Absence of retractions	Retraction of surrounding tissues

* With peripheral expansions. SUBC: subcutaneous tissue; DHI: dermo-hypodermal interface.

## Data Availability

Data are contained within the article.
